# Characterization of changes of pain behavior and signal transduction system in food-deprived mice

**DOI:** 10.1080/19768354.2018.1490348

**Published:** 2018-06-28

**Authors:** Sang-Pil Jang, Seong-Hwan Park, Jun-Sub Jung, Hee-Jung Lee, Jung-Woo Hong, Jae-Yong Lee, Hong-Won Suh

**Affiliations:** a Department of Pharmacology, Institute of Natural Medicine, College of Medicine, Hallym University, Chuncheon, Korea; b Department of Life science, Hallym University, Chuncheon, Korea; c Department of Biomedical Science, Hallym University, Chuncheon, Korea; d Department of Biochemistry, College of Medicine, Hallym University, Chuncheon, Korea

**Keywords:** Nociception, pain, food deprivation, signal transduction, dorsal root ganglia

## Abstract

Fasting in general causes several metabolic changes. In the present study, we examined the possible changes of several types of nociception during the food deprivation were investigated in mice. After the mice were forced into the fasting for 12, 24, or 48 h, the changes of nociception were measured by the tail-flick, writhing, formalin or von-frey tests. We found that the nociceptive behavior induced by intraperitoneally (i.p.) administered acetic acid (writhing response) or intraplantar injection of 5% formalin into the hind-paw were reduced in fasted group. In addition, the tail-flick response and threshold for nociception in mechanical von-frey test were also elevated in fasted group. Moreover, the p-CREB and p-ERK levels in the dorsal root ganglia (DRG) and the spinal cord were reduced in food-deprived group. Furthermore, p-AMPKα_1_ expressions in DRG and the spinal cord were up-regulated, whereas p-mTOR in DRG and the spinal cord was down-regulated in food-deprived group. Our results suggest that the chemical, mechanical, and thermal nociceptions appear to be reduced in a food-deprived mouse group. Additionally, reduction of nociception in food-deprived group appears to be closely associated with the expressions of several signal transduction molecules such as ERK, CREB, AMPKα_1_ and mTOR proteins in DRG and the spinal cord.

## Introduction

The nociception can be regulated by fasting. Earlier studies have demonstrated that the analgesia is produced by food deprivation (Hamm et al. 1985; Hamm and Knisely 1986). In addition, Davidson et al. (1992) have previously reported that the analgesia is developed by fasting for 24 h as revealed in the tail-flick test.

Both CREB and ERK proteins have been implicated in various types of pain transmission. For example, when nociceptive pain is stimulated, both CREB and ERK proteins expressions are elevated in dorsal root ganglia (DRG) and the spinal cord levels. The study (Crown et al. 2006) have demonstrated that the expression of spinal ERK and CREB proteins are well correlated with the allodynia development in spinal cord injury animal model. Han et al. (2011) have previously reported that ERK protein plays an important role in the production of neuropathic pain. In addition, both CREB and ERK protein expressions are elevated in neuropathic pain model, diabetic neuropathy model or capsaicin treated pain model (Miyabe and Miletic 2005; Song et al. 2005; Wu et al. 2005; Dang et al. 2014). However, the possible roles of CREB and ERK proteins in DRG and the spinal cord in the regulation of nociceptive changes in a food depravation animal model are not clear yet.

Several lines of evidence have demonstrated that the AMPK and mTOR systems are also closely related with nociception. For example, the expression of mTOR protein in DRG or the spinal cord are altered in several types pain models such as neuropathic pain (Zhang et al. 2013; Cui et al. 2014), diabetic neuropathy (He et al. 2016), and inflammation-induced pain (Asante et al. 2009; Xu et al. 2011) models. In addition, the activation of AMPK signal in general reduces nociception, allodynia or hyperalgesia observed in several types of pain models (Russe et al. 2013; Ma et al. 2015; Song et al. 2015; Hasanvand et al. 2016), suggesting that AMPK is an ultimate target molecules for relieving pain (Melemedjian et al. 2011; Asiedu et al. 2016; Price et al. 2016). However, the relationship between nociception and AMPK and mTOR systems during the food deprivation is still uncertain.

Although previous studies have demonstrated that pain perception during the fasting state is changed, the exact alteration of pain behavior and the mechanisms involved in the regulation of nociception during food deprivation have not been well characterized yet. Thus, the present study was designed to assess the possible pain behavior changes during food deprivation in several mouse pain models. Especially, the possible roles of ERK, CREB, AMPK, and mTOR proteins in the regulation of nociception during the food deprivation were investigated.

## Materials and methods

### Experimental animals

Male ICR mice (6 week of age) weighing 25–30 g at the beginning of experiments (Koatech, Seoul, Korea) were used in the experiments. The mice were housed 5 per cage in a room maintained at 22 ± 1°C with an alternating 12 h light-dark cycles. Food and water were available ad libitum. The animals were allowed to adapt to the laboratory for at least 2 hr before testing and were only used once. To reduce variation, all experiments were performed during the light phase of the cycle (10:00∼17:00).

### Nociceptive models and pain behavior measurements

For the visceral pain model (Koster et al. 1959), 1% acetic acid was injected intraperitoneally. The number of writhing response was counted for 30 min after acetic acid injection. For the formalin pain model (Hunskaar et al. 1985; Hunskaar and Hole 1987), 10 μl of 5% formalin was injected subcutaneously under the plantar surface of the left hind-paw. The pain behaviors such as shaking and licking the hind-paws were counted during the first (0–5 min) and the second (20–40 min) phases using a stop watch. The mechanical allodynia was assessed by von-frey tests (Bonin et al. 2014). For the von-frey test, mice were individually placed in a clear glass cells with a metal mesh floor allowed to adapt to the testing environment for 30 min, and then von-frey filaments (North Coast Medical, Inc., Gilroy, CA, USA) were applied to the plantar surface using an up and down paradigm. The number of animal used in the experiment was 8 in each group.

### Protein extraction and western blotting

The DRG and spinal cord of mice was dissected. Tissue was washed two times with cold Tris-buffered saline (20 mmol/L Trizma base and 137 mmol/L NaCl, pH 7.5). Immediately after washing, tissues were lysed with sodium dodecyl sulfate lysis buffer (62.5 mmol/L Trizma base, 2% w/v sodium dodecyl sulfate, 10% glycerol) containing 0.1 mmol/L Na3VO4, 3 mg/mL aprotonin, and 20mmolL NaF. After brief sonication, the concentration of protein was determined with a detergent-compatible protein assay reagent (Bio-Rad Laboratories, Hercules, CA, USA) using bovine serum albumin as the standard. After adding bromophenol blue (0.1% w/v), the proteins were boiled, separated by electrophoresis in 6%–10% polyacrylamide gels, and transferred onto the polyvinylidene difluoride membrane (Millipore, Bedford, MA, USA). The membranes were immunoblotted with antibodies p-AMPKα_1_ (Santa Cruz, 1:1000), p-mTOR (Abcam, 1:1000), p-ERK1/2 (Abcam, 1:1000), p-CREB (Abcam, 1:1000) and β-actin (Cell Signaling Technology, 1:1000) in blocking buffer for overnight. Membranes were then washed 4 times with Tris-buffered saline containing 20% Tween-20 (TBST; 10 mM Trizma base, pH8.0, 150 mM NaCl, and 20% Tween 20) for 20 min and then incubated with the anti-rabbit IgG-horseradish peroxidase conjugate (1:4000) in blocking buffer at room temperature for 1 h. After washing the membranes with TBST for 20 min (4 times), ECL-plus solution (Millipore, Billerica, MA, USA) was added. The membranes were then exposed to Luminescent Image Analyzer (LAS-4000, Fuji Film Co., Japan) for the detection of light emission. The specific signals were quantified with the Multi-Gauge Version 3.1 (Fuji Film, Japan) and expressed as the percentage of the control.

### Statistics

Statistical analysis was carried out by one-way analysis of variance (ANOVA) with a Bonferroni post-hoc test using GraphPad Prism Version 6.0 for Windows (GraphPad Software, USA). *P* values less than 0.05 were considered to indicate statistical significance. All values were expressed as the mean ± S.E.M.

## Results

### Changes of pain behaviors during food deprivation in several pain models

**Figure 1.**
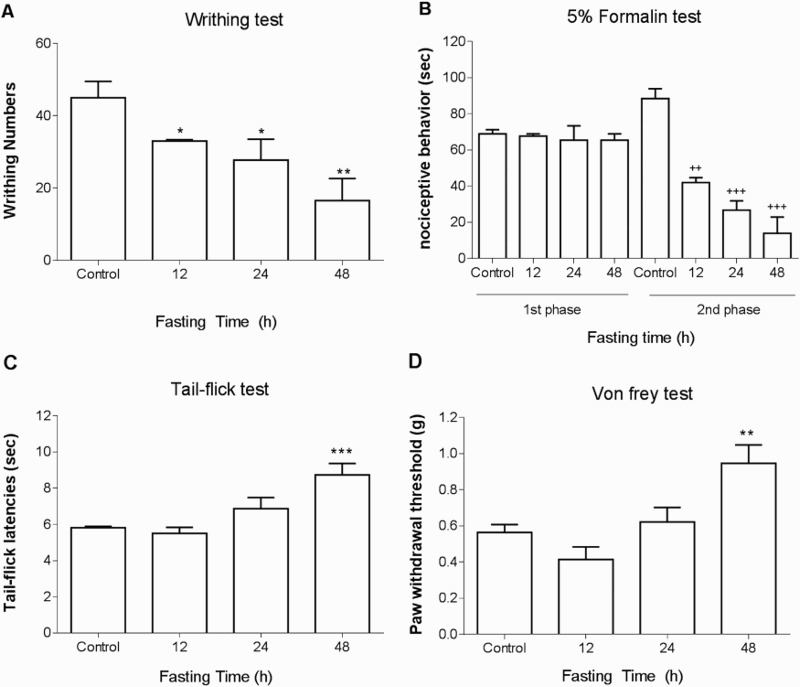
Nociceptive behavioral changes in food deprived group in various types of pain models. The mice were forced into food deprivation for 12, 24, or 48 h. Then, alteration of pain behaviors induced by (A) 1% acetic acid (i.p.), (B) 5% formalin (intraplantar injection into the hind-paw), (C) tail-flick or (D) mechanical pain stimulation by von-frey were assessed. The number of writhing response was counted for 30 min after acetic acid injection. In the formalin pain test, the pain behaviors such as vigorous licking and shaking paws were counted during the first (0–5 min) and the second (20–40 min) phases using a stop watch. The response time of tail-flick to radiant heat was measured. The mechanical pain threshold was measured by von-frey. The vertical bars indicate the standard error of mean (*P < 0.05, **p < 0.01, ***p < 0.001 compared to Control group. ++*P* < 0.001, +++*P *< 0.0001; compared to Control group). The mice number of animal used in each group was 8.

ICR mice were forced into fasting state for 12, 24, or 48 h, and the response to various types of pain stimulation was measured. In the writhing test, 1% acetic acid was administered i.p. and the number of writhing was counted for 30 min. As shown in Figure 1(A), the number of writhing response induced by acetic acid was reduced by food deprivation. In the formalin test, 5% formalin was injected into the hind-paw. As shown in Figure 1(B), the pain behaviors during both 1st and 2nd phases were reduced by food deprivation. Moreover, the tail-flick response was lengthened in food deprivation (Figure 1(C)). Furthermore, as shown in Figure 1(D), an elevation of threshold to mechanical stimulation was elevated as manifested by Von-frey filaments test.

### Changes of phosphorylated CREB, ERK, AMPK, and mTOR proteins in the dorsal root ganglia and the spinal cord during food deprivation

**Figure 2.**
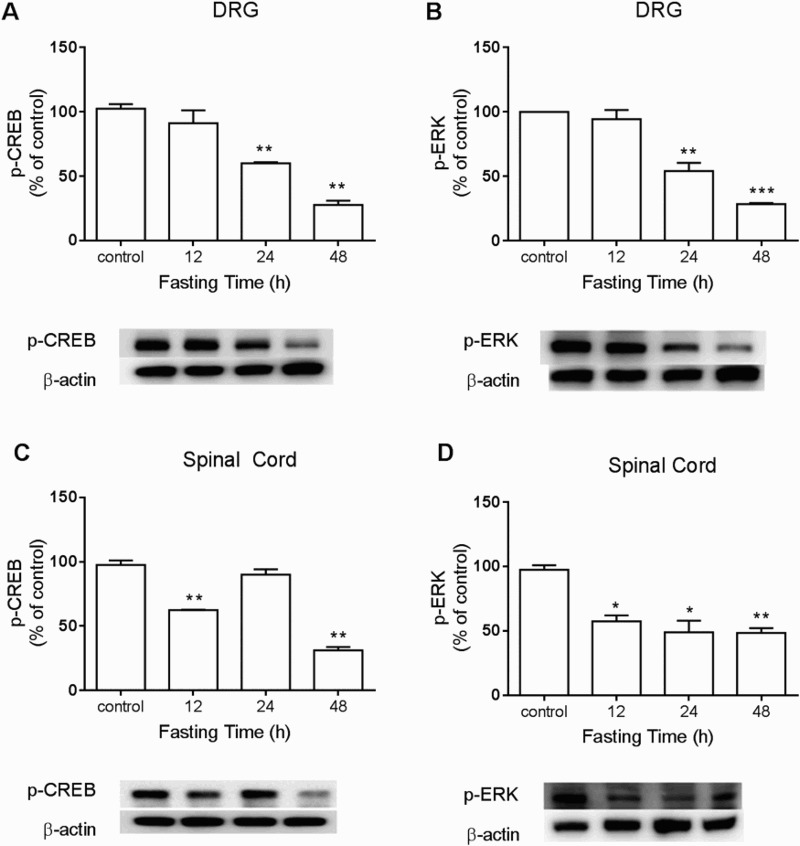
The changes of p-CREB and p-ERK expressions during food deprivation in the dorsal horn and the spinal cord. The mice were forced into food deprivation for 12, 24, or 48 h. Then, DRG and the lumbar region of the spinal cord were dissected. p-CREB and p-ERK expressions in the spinal cord and the DRG were analyzed by Western blot. The number of animals in each group was 6. β-Actin (1:1000 dilution) was used as an internal loading control. Signals were quantified with the use of laser scanning densitometry and expressed as a percentage of the control. Values are mean ± SEM (*P < 0.05, **P < 0.01, ***P < 0.001; compared to Control group).

**Figure 3.**
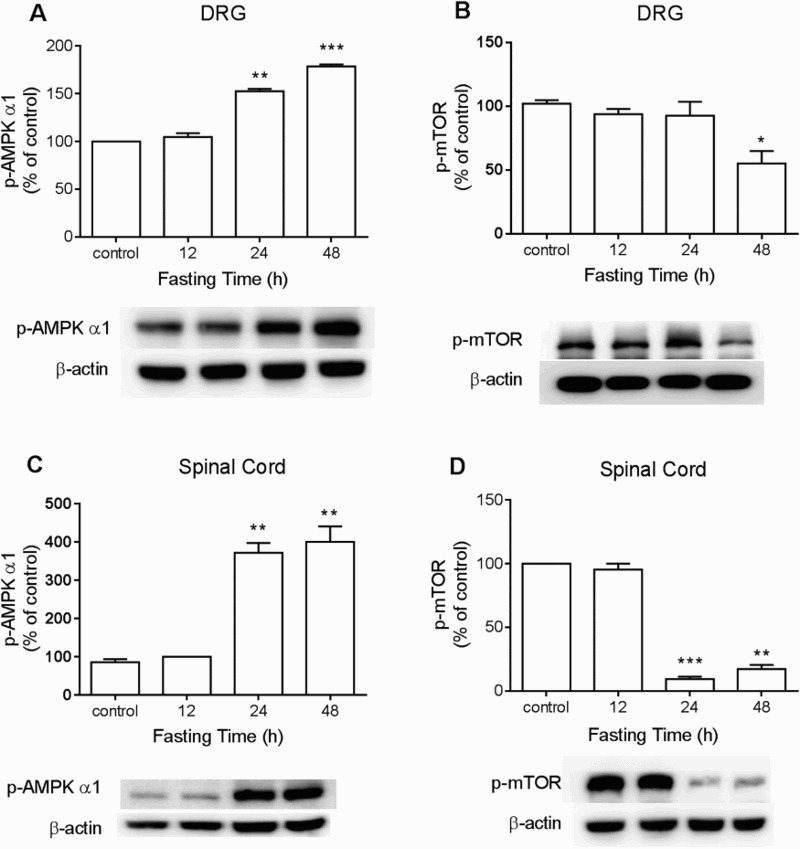
The changes of p-mTOR and p-AMPKα1 expressions during food deprivation in the dorsal horn and the spinal cord. The mice were forced into food deprivation for 12, 24, or 48 h. Then, DRG and the lumbar region of the spinal cord were dissected. p-mTOR and p-AMPKα1 expressions in the spinal cord and the DRG were analyzed by Western blot. The number of animals in each group was 6. β-Actin (1:1000 dilution) was used as an internal loading control. Signals were quantified with the use of laser scanning densitometry and expressed as a percentage of the control. Values are mean ± SEM (*P < 0.05, **P < 0.01, ***P < 0.001; compared to Control group)

To detect changes of CREB, ERK, AMPK, or mTOR expression in DRG and the spinal cord during food deprivation, the proteins were extracted from dissected DRG or lumbar spinal cord at 12, 24, or 48 h after food deprivation for Western blot analysis. As shown in Figure 2(A) and 2(B), food deprivation caused down-regulation of p-CREB and p-ERK protein levels in both DRG and the spinal cord. In addition, p-AMPKα_1_ expression in DRG and the spinal cord was up-regulated, whereas p-mTOR in DRG and the spinal cord was down-regulated in food deprivation group. (Figure 3(A) and 3(B)).

## Discussion

In the present study we found that the nociceptive behaviors were changed in several types of pain models in food deprived mouse group, such as writhing (chemical and visceral), formalin (chemical-induced inflammation), tail-flick (thermal), and von-frey (mechanical) pain models. The pain behaviors were reduced in food deprivation group in writhing and formalin pain models. In addition, the tail-flick response to radiant heat and threshold for mechanical pain were elevated in food deprived group, as revealed in tail-flick and von-frey stimulation tests, respectively, suggesting that the fasting may be closely related with the regulation of nociception. Our present findings are in line with several previous studies which demonstrated the food deprivation causes the analgesia in several pain models deprivation (Hamm et al. 1985; Hamm and Knisely 1986; Davidson et al. 1992). Furthermore, in the present study, we found the threshold to mechanical pain is also elevated in food deprivation group for the first time, suggesting that the mechanical nociception is also altered during fasting status.

Several lines of evidence have demonstrated that CREB and ERK proteins are closely associated with pain transmission. Both p-CREB and p-ERK expressions in the spinal cord or dorsal root ganglia are up-regulated in various types of chronic pain models, such as neuropathic pain and neuropathy (Miyabe and Miletic 2005; Song et al. 2005). Furthermore, p-CREB and p-ERK expressions in the spinal cord or brain regions are up-regulated in an acute inflammatory pain model such as formalin pain model (Hermanson and Blomqvist 1997; Seo et al. 2008; Hagiwara et al. 2009; Mao et al. 2013; Tochiki et al. 2015). In the present study, we found that down-regulation of p-CREB and p-ERK expressions in DRG and spinal cord were observed in food-deprived group, suggesting that the reduction of nociception in food-deprived group at least due to the reductions of p-CREB and p-ERK levels in the DRG and the spinal cord. In contrast to our results, several previous studies have reported that the phosphorylation of ERK and CREB are up-regulated in hypothalamic arcuate nucleus during fasting, while the expression of ERK in hypothalamic paraventricular nucleus is down-regulated during fasting (Shimizu-Albergine et al. 2001; Morikawa et al. 2004). However, in db/db mouse, phosphorylation of ERK and CREB are down-regulated in hypothalamic arcuate nucleus during fasting (Morikawa et al. 2004), indicating that the CREB or ERK proteins are differentially regulated in distinct regions in the central nervous system, depending on the energy balance.

In addition to p-CREB and p-ERK proteins, we also found that p-mTOR expressions in DRG and the spinal cord are down-regulated during the food deprivation up to 48 h, suggesting that the reduction of phosphorylation of mTOR might be responsible for the production of antinociception in food deprivation. Our findings are consistent with previous studies in the mTOR system in the activation of chronic pain models such as chronic inflammatory pain (Liang et al. 2013) or neuropathic pain (Asante et al. 2010), suggesting that mTOR system is activated during the elevation of pain perception. In addition, the strategy of reduction of mTOR system is one of new target for relieving the pain.

In contrast to the mTOR, we found that p-AMPKα_1_ expression in DRG and the spinal cord are up-regulated, suggesting that the increase of phosphorylation of AMPKα_1_ might be also responsible for the production of antinociception observed in food-deprived group. The activation of AMPK signal is generally associated with the regulation of nociception, allodynia or hyperalgesia observed in several types of pain models (Yamada et al. 1967; Russe et al. 2013; Song et al. 2015; Hasanvand et al. 2016). To support of these findings, the AMPK activators such as resveratrol and metformin exert an antinociceptive effect in chronic pain models (Tillu et al. 2012; Ma et al. 2015).
